# Comparison of biomechanical assessment between upright and supine inspiratory-expiratory area-detector CT in progressive pulmonary fibrosis

**DOI:** 10.1186/s41747-026-00698-y

**Published:** 2026-03-31

**Authors:** Yoshiharu Ohno, Kota Aoyagi, Yoshiyuki Ozawa, Masahiko Nomura, Hirona Kimata, Yuya Ito, Kenji Fujii, Takahiro Ueda, Junichiro Araoka, Naruomi Akino, Takeshi Yoshikawa, Daisuke Takenaka, Masahiko Endo, Yasushi Hoshikawa, Hidekata Yasuoka, Tomoya Horiguchi, Yasuhiro Goto, Naozumi Hashimoto, Kazuyoshi Imaizumi

**Affiliations:** 1 Department of Diagnostic Radiology, Fujita Health University School of Medicine, Toyoake, Japan; 2 Joint Research Laboratory of Advanced Medical Imaging and Artificial Intelligence, Fujita Health University School of Medicine, Toyoake, Japan; 3 Canon Medical Systems Corporation, Otawara, Japan; 4 Division of Diagnostic Radiology, Shizuoka Cancer Center, Nagaizumi-cho, Sunto-gun, Shizuoka, Japan; 5 Department of Thoracic Surgery, Fujita Health University School of Medicine, Toyoake, Japan; 6 Division of Rheumatology, Department of Internal Medicine, Fujita Health University School of Medicine, Toyoake, Japan; 7 Department of Respiratory Medicine, Fujita Health University School of Medicine, Toyoake, Japan

**Keywords:** Biomechanical phenomena, Lung diseases (interstitial), Pulmonary fibrosis, Respiratory function tests, Tomography (x-ray computed)

## Abstract

**Background:**

We compared the capabilities of quantitatively assessed paired inspiratory-expiratory area-detector computed tomography (ADCT) for pulmonary functional loss and disease severity evaluations between upright and supine ADCT in matched progressive pulmonary fibrosis (PPF) patients.

**Materials and methods:**

This retrospective cohort consisted of age-, sex-, and underlying disease-matched patients with PPF who underwent paired inspiratory-expiratory CT on upright ADCT (*n* = 40) and supine ADCT (*n* = 40), pulmonary function tests, and disease severity assessment. Based on CT data, the absolute values of the logarithm of the Jacobian determinant and warp-field magnitude of the whole lung and all lobes were calculated. Stepwise regression analyses were performed.

**Results:**

On supine ADCT, both indices of the left lower lobe (LLL) were the first and only steps for pulmonary function test results and CT-assessed disease severity (absolute value of the logarithm of the Jacobian determinant: 0.139 ≤ *r*^2^ ≤ 0.175, 0.007 ≤ *p* ≤ 0.018; absolute value of the warp-field magnitude: 0.371 ≤ *r*^2^ ≤ 0.447, *p* < 0.001). However, on upright ADCT, both indices indicated that LLL was the first step and the right lower lobe was the second step for pulmonary function test results and CT-assessed disease severity (0.503 ≤ *r*^2^ ≤ 0.674, *p* < 0.001 or 0.000 < *p* ≤ 0.006 and 0.474 ≤ *r*^2^ ≤ 0.652, 0.002 ≤ *p* ≤ 0.045, respectively).

**Conclusion:**

Upright ADCT has equal to or better potential than supine ADCT for detecting pulmonary functional loss and evaluating disease severity when paired inspiratory-expiratory ADCT is applied in PPF patients.

**Relevance statement:**

Upright ADCT has superior potential to supine ADCT for pulmonary functional loss and disease severity evaluations when paired inspiratory-expiratory ADCT is performed in patients with progressive pulmonary fibrosis (PPF).

**Key Points:**

- Matched progressive pulmonary fibrosis patients compared functional loss and disease severity evaluations between inspiratory-expiratory upright and supine area-detector CT.
- Clinical parameters demonstrated better correlations with upright than with supine inspiratory-expiratory area-detector CT.
- Warp-field magnitude showed better correlations with disease severities than the logarithm of the Jacobian determinant on each area-detector CT.

**Graphical Abstract:**

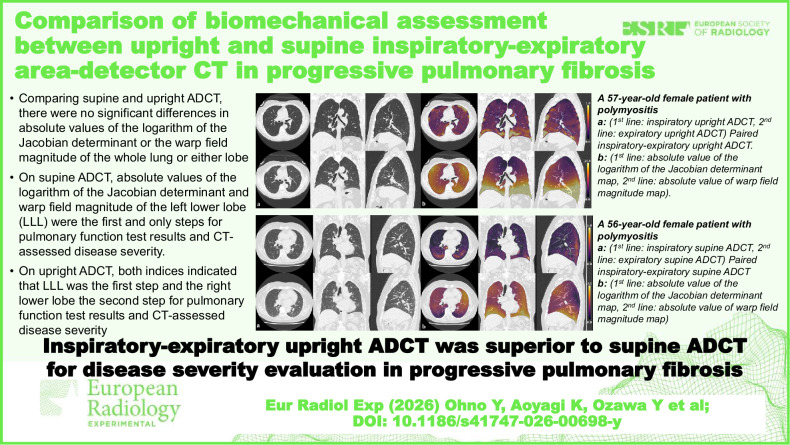

## Background

Fibrosing interstitial lung disease (ILD) encompasses a diverse array of conditions, such as idiopathic pulmonary fibrosis (IPF), connective tissue disease-related ILD, fibrotic hypersensitivity pneumonitis, and occupational lung disorders. It is associated with varying degrees of morbidity and mortality.

IPF is often regarded as the essential illustration of progressive fibrotic ILD [1]. Several other fibrotic ILDs can progress, including sarcoidosis, hypersensitivity pneumonitis, and connective tissue disease-related ILD. Although precise data on the percentage of progressive pulmonary fibrosis (PPF) in patients with non-IPF are lacking, estimates suggest that it affects approximately 13‒60% of individuals with non-IPF ILD [2–4]. Originally defined as progressive fibrosing ILD [5–7], an official American Thoracic Society (ATS), European Respiratory Society (ERS), Japanese Respiratory Society (JRS), and Associatión Latinoamericana de Tórax (ALAT) (ATS/ERS/JRS/ALAT) clinical practice guideline published in 2022 defined PPF as non-IPF fibrosing ILD with the presence of two of the following three features occurring within a year of follow-up: deterioration in respiratory symptoms demonstrable physiological evidence of disease progression through pulmonary function tests, or increased fibrosis on imaging [4]. Although thin-section computed tomography (CT) is widely used to assess not only PPF or progressive fibrosing ILD but also other ILDs, additional quantitative radiological imaging is needed to evaluate lung structure changes in these patients.

Upright area-detector CT (ADCT) was developed by Canon Medical Systems and has been tested over the last several years [8–10]. Although upright ADCT was suggested to be comparable to supine ADCT in terms of physical characteristics, upright ADCT offers substantial advantages as follows: (1) potential to replace poorer quality x-ray examinations; (2) visualize cross sections of the entire human body with gravity; and (3) potential to replace all pulmonary functional CT methods. Furthermore, volume-based evaluations on upright or sitting ADCT had slightly better correlations than supine ADCT with pulmonary functional test results in volunteers and patients with chronic obstructive pulmonary disease or ILD [8–10]. In addition, no study has compared the results of entire- and lobar-based biomechanical evaluations from paired inspiratory-expiratory ADCT data for pulmonary functional loss assessment and disease severity evaluations between upright and conventional supine ADCT in patients with PPF.

We thus hypothesized that upright ADCT had better potential than supine ADCT for evaluating pulmonary functional loss and disease severity from paired inspiratory and expiratory ADCT in patients with PPF. This study directly compared the capabilities of quantitatively assessed paired inspiratory and expiratory ADCT data for pulmonary functional loss and disease severity evaluations between upright and supine ADCT in matched patients with PPF.

## Materials and methods

This retrospective study was approved by the institutional review board of Fujita Health University Hospital. This study was also compliant with the Health Insurance Portability and Accountability Act, which waived the requirement for written informed consent, and was technically and financially supported by the Canon Medical Systems Corporation and Smoking Research Foundation. In each participant, informed consent was obtained in the form of opt-out on the website, and those who rejected were excluded from this study.

This study was financially and technically supported by the Canon Medical Systems Corporation. Six of the authors are employees of Canon Medical Systems (K.A., H.K., Y.I., K.F., J.A., and N.A.) and did not have control over any of the data used in this study and information submitted for publication or over which data and information were to be included in this study. All data are controlled by staff in Fujita Health University School of Medicine and Hospital (Y.O., Y.Oz., M.N., T.U., T.Y., D.T., M.E., Y.H., H.Y., T.H., Y.G., N.H., and K.I.). All statistical analyses were performed by Department of Diagnostic Radiology staff in Fujita Health University School of Medicine and Hospital (Y.O., Y.Oz., M.N., T.U., T.Y., and D.T.).

### Subjects

**Fig. 1:**
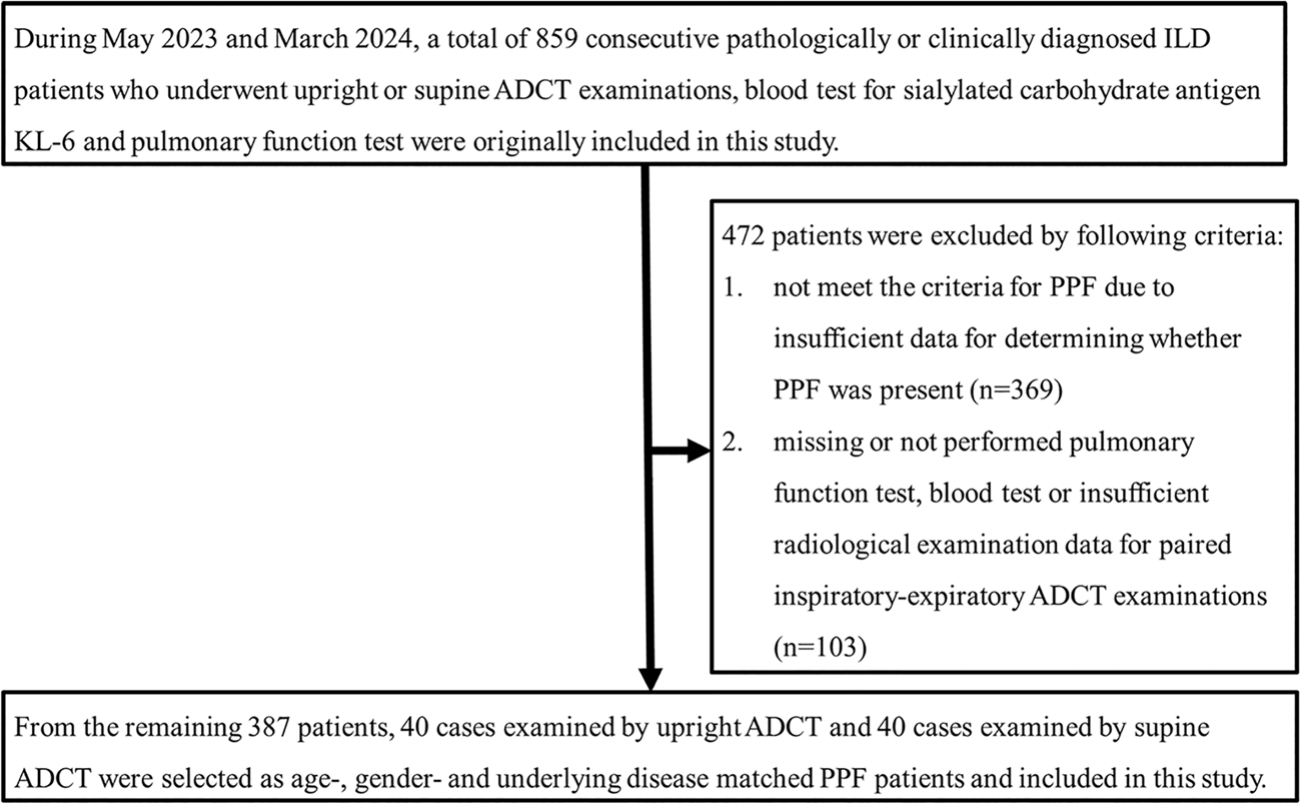
Patient selection flow diagram

Between May 2023 and March 2024, a total of 859 consecutive pathologically or clinically diagnosed ILD patients who underwent upright or supine ADCT examinations, blood tests for sialylated carbohydrate antigen KL-6 (KL-6), and pulmonary function tests were originally included in this study. In accordance with previously published guidelines [4, 5], patients with PPF were selected to meet either the recent criteria proposed by the 2022 Clinical Practical Guidelines [4] or the criteria for progressive fibrosing ILD proposed in the INBUILD trial [5]. The guideline criteria for PPF require at least 2 of the following to occur within the previous 12 months: (1) worsening respiratory symptoms; (2) an absolute decline in FVC ≥ 5% predicted or an absolute decline in diffusing capacity for carbon monoxide (DL_CO_) ≥ 10% predicted; or (3) radiological evidence of disease progression. The progressive fibrosing ILD criteria [5] require meeting at least 1 of the following within the previous 24 months: (1) a relative decline in forced vital capacity (FVC) ≥ 10% predicted; (2) a relative decline in FVC ≥ 5% to < 10% predicted and worsening respiratory symptoms or an increased extent of fibrosis on thin-section CT; or (3) worsening of respiratory symptoms and an increased extent of fibrosis. All pulmonary functional tests were performed within 1 week (mean: 1.2 days; range: 1‒4 days) after paired inspiratory-expiratory ADCT examinations. Figure 1 shows the flow diagram of this study.

### CT protocol

All paired inspiratory-expiratory ADCT examinations were performed using a 320-detector-row upright ADCT (TSX-401R; Canon Medical Systems) or two 320-detector-row supine ADCT (Aquilion ONE/PRISM or INSIGHT Edition; Canon Medical Systems) without contrast media administration. All inspiratory-expiratory upright ADCT data were obtained at the standing position in this study. On each paired inspiratory-expiratory ADCT examination, the following scan parameters were applied: detector collimation, 0.5 mm × 80 rows; tube voltage, 120 kVp; tube current, automatic exposure control with the noise index determined by the image standard deviation as 13; pitch factor, 0.813; rotation time, 0.5 s/rotation; reconstruction section thickness, 1 mm; acquisition and reconstruction matrix, 512 × 512; volume CT dose index, 13.0 mGy for supine ADCT and 12.6 mGy for upright ADCT; and dose length product, 496.8 mGy·cm for supine ADCT and 495.7 mGy·cm for upright ADCT. All CT images were reconstructed using hybrid-type iterative reconstruction (Adaptive Iterative Dose Reduction 3D; Canon Medical Systems) with standard lung kernels (FC52; Canon Medical Systems). The scan protocol of inspiratory-expiratory supine ADCT had been fixed and performed since January 2023, and that of inspiratory-expiratory ADCT was fixed and performed since May 2023. Details of the scan protocol for upright and supine ADCT are shown in Table 1.

**Table 1:** Supine and upright ADCT protocols in this study

	Supine ADCT	Upright ADCT
CT system	320-detector-row CT (Aquilion ONE/PRISM or INSIGHT Editions)	320-detector-row CT (TSX-401R)
Scan method	Helical scan	
Detector collimation	0.5 mm × 80 rows	
Beam pitch	0.813	
Tube voltage (kVp)	120	
Tube current (mA)	auto mA (image standard deviation (SD) = 13)	
Rotation time (s/rotatiob)	0.5	
Field of view (mm)	240 × 240‒400 × 400	
Slice thickness (mm)	1	
Reconstruction method	Hybrid-type iterative reconstruction (AIDR 3D)	
Reconstruction matrix	512 × 512	
Volume CT dose index (mGy)	13.0	12.6
Dose length product (mGy·cm)	496.8	495.7

*ADCT* Area-detector computed tomography, *AIDIR 3D* Adaptive iterative dose reduction 3D

### Clinical information

The following clinical information was obtained by searching electronic medical records: age, sex, clinical diagnosis of ILD, and pulmonary function test results obtained within a few weeks (7 ± 2 days) of the CT examination. For each patient, the percentage of vital capacity (%VC), percentage of forced vital capacity (%FVC), percentage of predicted forced expiratory volume in 1 s (%FEV_1_), and percentage of diffusion capacity of the lung for carbon monoxide (%DL_CO_) were assessed according to the American Thoracic Society guidelines [11–13]. The FVC was expressed as the volume and percentage (%) of the predicted normal values.

### Quantitative assessments of lung biomechanics as evaluated by paired inspiratory-expiratory ADCT

**Fig. 2:**
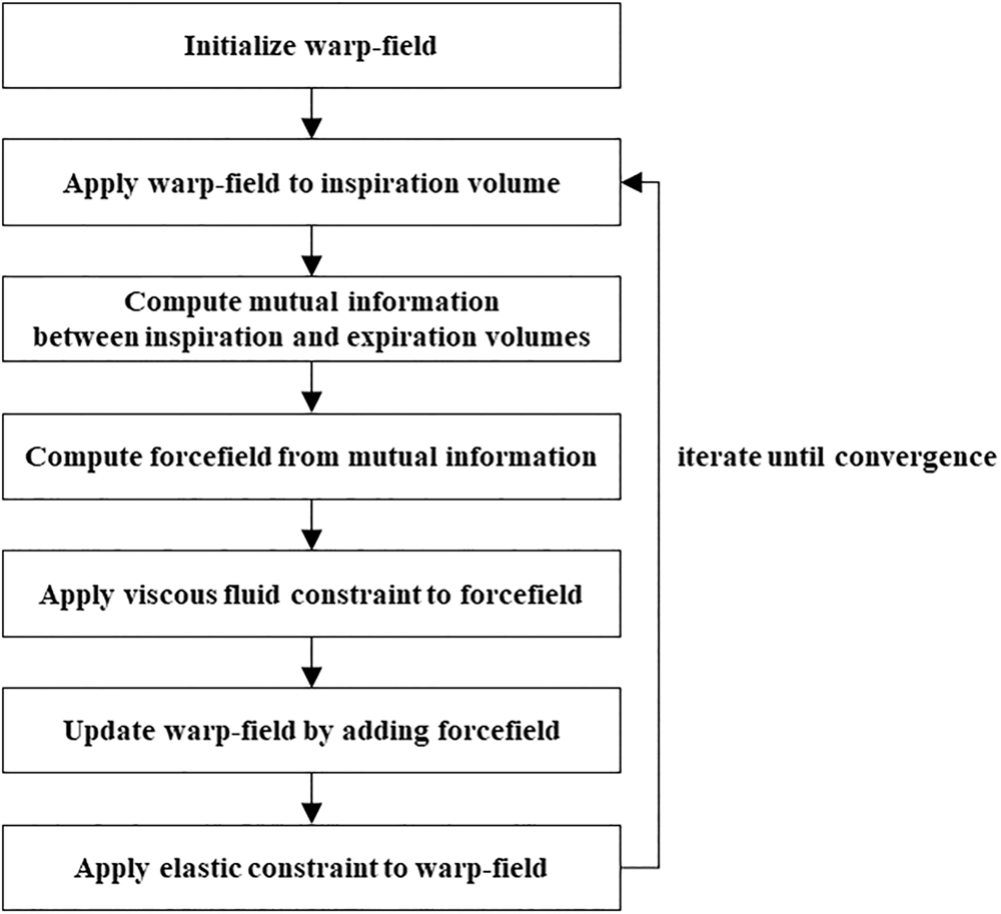
Shame for quantitative biomechanical analysis from paired inspiratory-expiratory computed tomography data. The processing workflow of the non-rigid registration of lung volumes between inspiration and expiration is shown. It is based on a mutual information force model and a multiscale system with simple fluid and elastic regularization. The warp field was iteratively updated until convergence, after which the final warp field was used to calculate the quantitative value of the logarithm of the Jacobian determinant and warp-field magnitude

Quantification of lung biomechanics was based on non-rigid registration of the lung volumes between inspiration and expiration using the mutual information force model derived by Crum et al [14] and the multiscale system with simple fluid and elastic regularization described by Thirion [15]. First, the initial mutual information between the inspiration volume as the reference volume and the expiration volume as the targeted (or floating) volume was computed. The force field, which is the gradient of the mutual information, was calculated using a finite-difference method, and the viscous fluid constraint was applied. The force field was then added to the current warp field, and an elastic constraint was applied to obtain the updated warp field. The viscous fluid constraint and elastic constraint were provided by Gaussian smoothing to obtain a smooth warp field. The updated warp field was applied to the floating volume to obtain the warped floating volume. New mutual information between the reference volume and the warped floating volume was computed. These steps were performed iteratively until convergence was achieved. The final warp field was used to quantify the lung biomechanical parameters. The logarithm of the Jacobian determinant represented the local volume change at the voxel level, and the voxel size after deformation is interpreted as unchanged if the value equals zero, as expansion if the value is positive, and as shrinkage if the value is negative. On the other hand, the warp-field magnitude represents the magnitude of the movement of each voxel between the inspiratory and expiratory CT volumes. The shame quantitative assessment of lung biomechanics is shown in Fig. 2.

### Image analysis

On quantitative image analyses for lung biomechanics assessments, all paired inspiratory-expiratory ADCT data were analyzed by a board-certified chest radiologist with 31 years’ experience (Y.O.) using a commercially available workstation (Vitrea; Canon Medical Informatics) with a proprietary lung biomechanics evaluation software program (Proto-Lung Motion Analysis, version 0.0.4; Canon Medical Systems).

To evaluate regional and entire lung motion on upright and supine ADCT, absolute values of the logarithm of the Jacobian determinant and the warp-field magnitude were computationally analyzed pixel-by-pixel. Both indices of each lobe and the whole lung were then automatically determined as the average value within the lobe and the entire lung, respectively, in each case.

For semiquantitative disease severity assessments for PPF, CT-assessed disease severity was independently scored by two other board-certified chest radiologists with 22- and 34-year experience (Y.Oz. and D.T.) using a picture archiving and communication system (PACS) (RapideyeCore TFS01; Canon Medical Systems), with the reviewers having no access to information about the technical parameters. Furthermore, both reviewers assessed disease severity without having access to any information on disease severity based on serum KL-6 levels or pulmonary function test results for any of the subjects. As previously described [16–19], the visual evaluation included both severity and extent scores. The severity is based on the assessment of parenchymal abnormalities assumed to reflect increasing severity of lung involvement, such as ground glass opacity (score = 1), irregular pleural margins (score = 2), septal and subpleural lines (score = 3), honeycombing (score = 4), and subpleural cysts (score = 5) [16–19]. The overall severity score ranged from 0 (no abnormality) to 15 (all abnormalities were present). The extent score was obtained by counting the bronchial pulmonary segments in which any of the previous abnormalities were observed: involvement of one to three segments rated a score of 1, involvement of four to nine segments a score of 2, and involvement of more than nine segments a score of 3. Thus, the overall extent score ranges from 0 (no abnormality in any segment) to 15 (all five abnormalities in more than nine segments). Severity and extent scores were then added to obtain the total disease score (range: 0–30) [16–19]. Finally, the CT-assessed disease severity for each patient was determined by the consensus of the two reviewers.

### Statistical analysis

To determine the difference between the two groups, each index was compared by χ^2^ test or Student’s *t*-test, and the intraclass correlation coefficient (ICC) was also calculated. To compare the difference in each index between supine and upright ADCT, absolute values of the logarithm of the Jacobian determinant and warp-field magnitude of each lobe and whole lung were compared using Student’s *t*-test. To assess the capability of pulmonary functional loss evaluation on each paired inspiratory-expiratory ADCT, Pearson’s correlations were performed between the indices of each lobe and the whole lung and the %VC, %FVC, %FEV_1_, %DL_CO_, serum KL-6 level, and CT-assessed disease severity. To determine the influence of lung biomechanics in all lobes on pulmonary function tests, serum KL-6 levels, and CT-assessed disease severity, and stepwise regression analyses were performed.

A *p*-value < 0.05 was considered significant for each statistical analysis. According to the past literature [20], correlation coefficient (*r*) was interpreted as follows: -0.2 < *r* < 0 or 0 < *r* < 0.2 as poor, -0.6 < *r* ≤ -0.2 or 0.2 ≤ *r* < 0.6 as fair, -0.8 < *r* ≤ -0.6 or 0.6 ≤ *r* < 0.8 as moderate, and -1 < *r* ≤ -0.8 or 0.8 ≤ *r* < 1.0 as very strong. All statistical analyses were performed using a statistical software (JMP version 14: SAS Institute Japan, StatMate III) or a free statistical software program (EZR version 1.54: https://www.jichi.ac.jp/saitama-sct/SaitamaHP.files/statmedEN.html) [21]. Moreover, EZR was used for 1:1 propensity score matching on age, sex, and underlying disease for selecting upright and supine ADCT groups in this study.

## Results

Of the original 859 patients, 369 were excluded because they did not meet the criteria for PPF due to insufficient data for determining whether or not PPF was present, and 103 out of 859 patients were excluded because pulmonary function tests, blood tests, or radiological examinations for paired inspiratory-expiratory ADCT examinations were missing data or not performed. Of the remaining 387 patients, 40 cases examined using upright ADCT and 40 cases examined using supine ADCT were selected after 1:1 propensity score matching on age, sex, and underlying disease and included in this study.

**Fig. 3:**
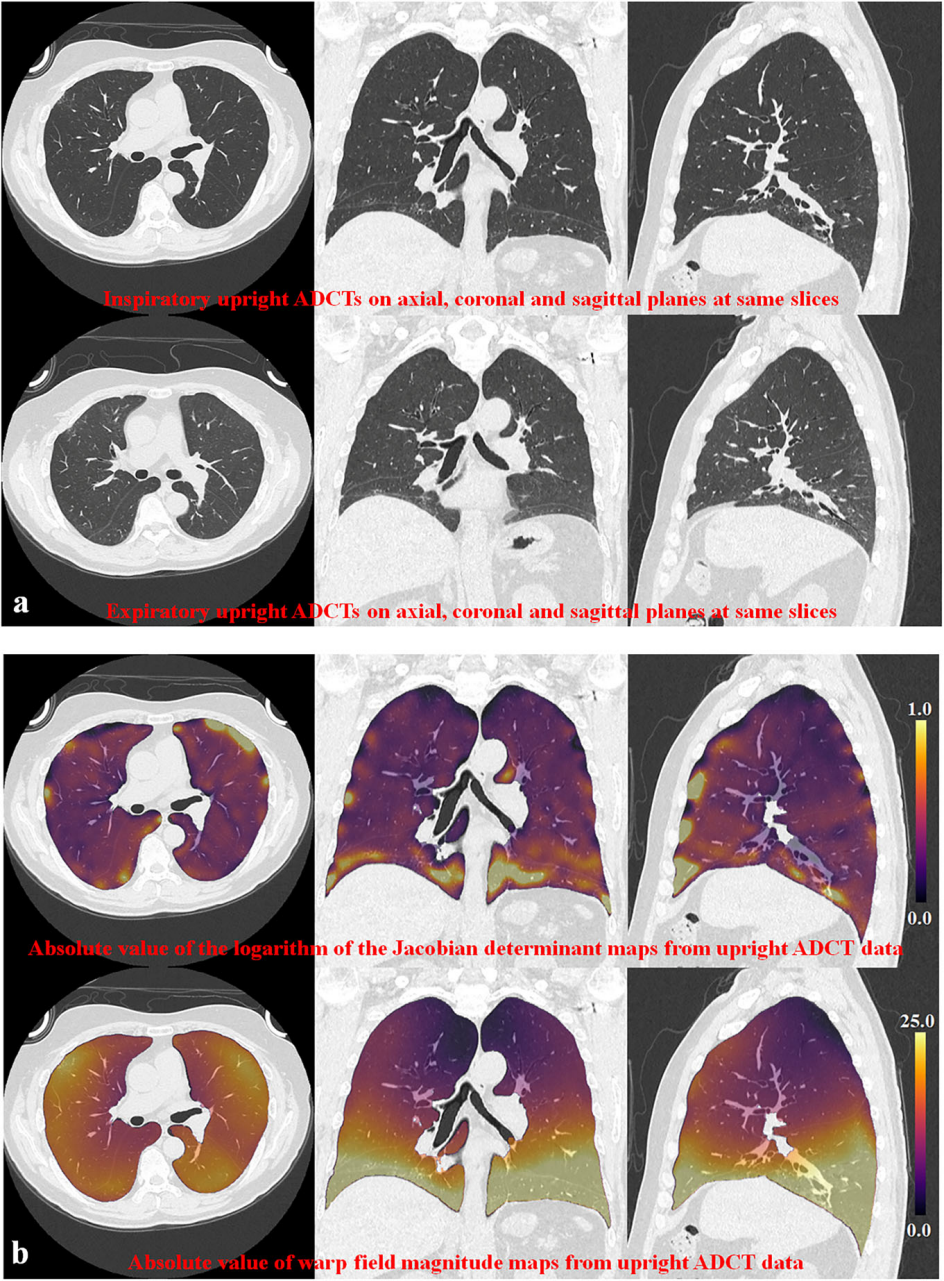
A 57-year-old female patient with polymyositis (%VC: 87%, %FVC: 89%, %FEV1: 81%, %DL_CO_: 84%, KL-6: 456U/mL, CT-assessed disease severity: 4). **a** 1st line: inspiratory upright ADCT; 2nd line: expiratory upright ADCT. Paired inspiratory-expiratory upright ADCT shows lung volume changes as well as lung shape, chest wall, and diaphragm positions. **b** 1st line: absolute value of the logarithm of the Jacobian determinant map; 2nd line: absolute value of the warp-field magnitude map. Both maps demonstrated larger absolute values of the logarithm of the Jacobian determinant and warp-field magnitude in the anterior than in the posterior lung areas, as well as the lower lung area being closer to the diaphragm than the upper lung area. *For acronyms, see the list of Abbreviations*.

**Fig. 4:**
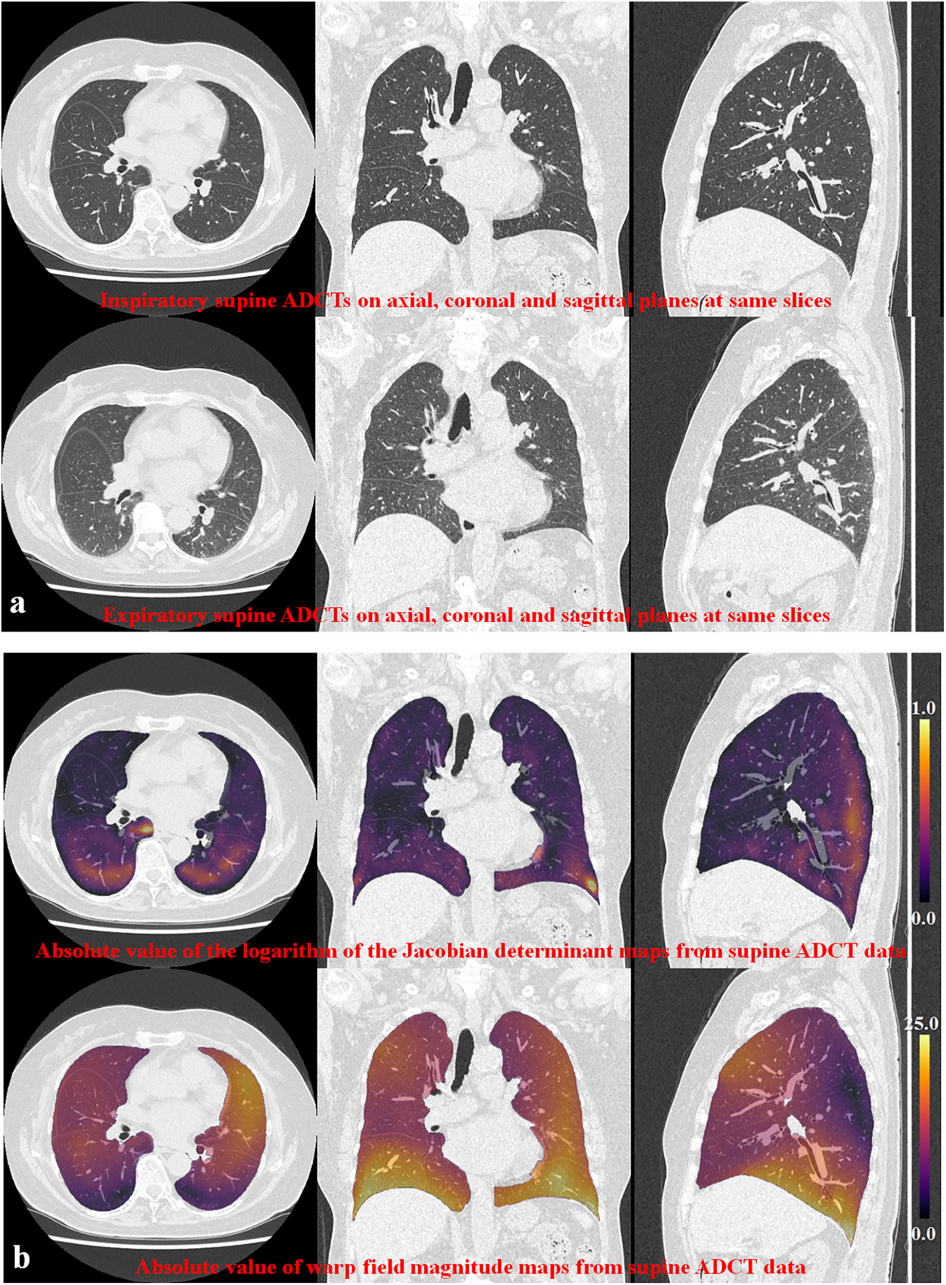
A 56-year-old female patient with polymyositis (%VC: 89%, %FVC: 85%, %FEV_1_: 83%, %DL_CO_: 87%, KL-6: 484U/mL, CT-assessed disease severity: 3). **a** 1st line: inspiratory supine ADCT; 2nd line: expiratory supine ADCT. Paired inspiratory-expiratory supine ADCT shows lung volume changes as well as lung shapes, chest walls, and diaphragm positions, although these changes are smaller than on upright ADCT. **b** 1st line: absolute value of the logarithm of the Jacobian determinant map; 2nd line: absolute value of the warp-field magnitude map. Both maps demonstrated smaller absolute values of the logarithm of the Jacobian determinant and warp-field magnitude in the posterior than in the anterior lung areas, as well as the lower lung area being closer to the diaphragm than the upper lung areas. Furthermore, both indices of supine ADCT were smaller than those of upright ADCT in each voxel. *For acronyms, see the list of Abbreviations*.

Representative cases for inspiratory-expiratory CTs on upright and supine ADCTs are shown in Figs. 3 and 4.

Patient characteristics are shown in Table 2. There were no significant differences in gender, age, body mass index, smoking history, comorbidity, pulmonary function test results, including %VC, %FVC, %FEV1 and %DL_CO_, serum KL-6 level, underlying diseases, or CT-assessed disease severity between the two groups (0.258 ≤ *p* ≤ 1.000). ICCs for CT-assessed disease severity on supine and upright ADCTs were determined as 0.997 and 0.998.

**Table 2:** Patients’ characteristics

			Upright ADCT	Supine *versus* upright ADCT *p-*value
Gender (cases)	Male	25	25	1.000
	Female	15	15	
Age (years)	Mean ± SD	70 ± 14	73 ± 10	0.366
	(range)	(42‒87)	(46‒89)	
Body mass index (kg/m²)	Mean ± SD	22.2 ± 3.4	21.8 ± 3.3	0.572
Smoking history (pack/year)	Mean ± SD	24.7 ± 20.7	24.2 ± 20.4	0.909
Comorbidity (cases)	Aortic aneurysm	5	3	0.679
	Cardiac dysfunction	1	2	
	Chronic liver dysfunction	4	3	
	Chronic renal dysfunction	5	3	
	Diabetes mellitus	8	4	
	Hypertension	4	9	
	Ischemic heart disease	4	3	
	Valvular heart disease	3	4	
	None	6	9	
%VC (%)	Mean ± SD	82.3 ± 34.2	87.4 ± 36.9	0.566
%FVC (%)	Mean ± SD	85.7 ± 36.3	91.5 ± 38.6	0.489
%FEV_1_ (%)	Mean ± SD	76.8 ± 31.1	80.0 ± 33.7	0.658
%DL_CO_ (%)	Mean ± SD	71.9 ± 30.0	74.0 ± 31.2	0.762
Serum KL-6 level (U/mL)	Mean ± SD	492.3 ± 269.1	470.4 ± 206.3	0.684
Underlying diseases (cases)	Idiopathic pulmonary fibrosis	7	8	0.996
	Progressive systemic sclerosis	6	7	
	Polymyositis	7	8	
	Dermatomyositis	7	7	
	Mixed connective tissue disease	6	5	
	Chronic hypersensitive pneumonitis	3	2	
	Sarcoidosis	4	4	
CT-assessed disease severity	Mean ± SD	8.3 ± 7.8	6.2 ± 8.7	0.258

*ADCT* Area-detector computed tomography, *KL-6* Sialylated carbohydrate antigen KL-6, *%DL*_*CO*_ Percentage of diffusion capacity of the lung for carbon monoxide, *%FEV*_*1*_ Percentage of predicted forced expiratory volume in 1 s, *%FVC* Percentage of forced vital capacity, *%VC P*ercentage of vital capacity *SD* Standard deviation

Table 3 shows a comparison of each index between supine and upright ADCT. There were no significant differences in absolute values of the logarithm of the Jacobian determinant or the warp-field magnitude of the whole lung or either lobe (0.083 ≤ *p* ≤ 0.998).

**Table 3:** Comparison of each index between supine and upright ADCT

Index	Whole lung or lobe	Supine ADCT	Upright ADCT	Supine *versus* upright ADCT
		Mean ± SD	Mean ± SD	*p*-value
Absolute value of the logarithm of the Jacobian determinant	Whole lung	-0.430 ± 0.391	-0.348 ± 0.331	0.313
	Left upper lobe	-0.304 ± 0.321	-0.204 ± 0.164	0.083
	Left lower lobe	-0.509 ± 0.367	-0.509 ± 0.533	0.998
	Right upper lobe	-0.282 ± 0.282	-0.156 ± 0.163	0.083
	Right middle lobe	-0.528 ± 0.838	-0.423 ± 0.590	0.517
	Right lower lobe	-0.526 ± 0.417	-0.446 ± 0.469	0.421
Absolute value of the warp-field magnitude	Whole lung	18.034 ± 8.634	16.106 ± 12.494	0.425
	Left upper lobe	19.054 ± 11.146	14.106 ± 14.659	0.093
	Left lower lobe	17.243 ± 8.300	17.479 ± 11.583	0.917
	Right upper lobe	17.708 ± 11.333	13.712 ± 15.215	0.187
	Right middle lobe	18.465 ± 10.764	17.035 ± 13.393	0.600
	Right lower lobe	17.701 ± 9.323	18.200 ± 13.046	0.844

*ADCT* Area-detector CT, *SD* Standard deviation

Correlations between both indices in the whole lung or each lobe and pulmonary function test results, serum KL-6 levels, and CT-assessed disease severity are shown in Table 4. Correlations of absolute values of the logarithm of the Jacobian determinant of whole lung and each lobe on supine ADCT, whole lung, both lower lobes, and right upper lobe had significantly fair correlations with pulmonary function test results, serum KL-6 level (-0.418 ≤ *r* ≤ -0.325, 0.000 < *p* ≤ 0.040), and CT-assessed disease severity (0.318 ≤ *r* ≤ 0.373, 0.018 ≤ *p* ≤ 0.045). In contrast, the indices of the whole lung and each lobe on upright ADCT had significantly fair or moderate correlations with pulmonary function test results, serum KL-6 level (-0.737 ≤ *r* ≤ -0.459, *p* < 0.0001 or 0.000 < *p* ≤ 0.003), and CT-assessed disease severity (0.393 ≤ *r* ≤ 0.657, *p* < 0.0001 or 0.000 < *p* ≤ 0.012). Regarding correlations of absolute values of the warp-field magnitude in the whole lung and each lobe on supine ADCT, the whole lung and each lobe had significantly fair or moderate correlations with pulmonary function test results, serum KL-6 level (0.381 ≤ *r* ≤ 0.668, *p* < 0.001 or 0.000 < *p* ≤ 0.015), and CT-assessed disease severity (-0.615 ≤ *r* ≤ -0.400, *p* < 0.001 or 0.000 < *p* ≤ 0.010). Furthermore, the indices of the whole lung and each lobe on upright ADCT had significantly fair, moderate or good correlations with pulmonary function test results, serum KL-6 level (0.373 ≤ *r* ≤ 0.783, *p* < 0.001 or 0.000 < *p* ≤ 0.018), and CT-assessed disease severity (-0.780 ≤ *r* ≤ -0.608, *p* < 0.001).

**Table 4:** Correlations between both indices in the whole lung or each lobe on supine and upright ADCT and pulmonary function test results, serum KL-6 levels, and CT-assessed disease severity

Pulmonary function test results, serum KL-6 levels, and CT-assessed disease severity	Index	Whole lung or lobe	Supine ADCT		Upright ADCT	
			*r*	*p*-value	*r*	*p*-value
Percentage of vital capacity	Absolute value of the logarithm of the Jacobian determinant	Whole lung	-0.288	0.072	-0.643	< 0.001
		Left upper lobe	-0.262	0.102	-0.609	< 0.001
		Left lower lobe	-0.398	0.011	-0.639	< 0.001
		Right upper lobe	-0.279	0.082	-0.568	< 0.001
		Right middle lobe	-0.151	0.354	-0.487	0.001
		Right lower lobe	-0.305	0.055	-0.640	< 0.001
	Absolute value of the warp-field magnitude	Whole lung	0.627	< 0.001	0.783	< 0.001
		Left upper lobe	0.451	0.004	0.602	< 0.001
		Left lower lobe	0.627	< 0.001	0.734	< 0.001
		Right upper lobe	0.406	0.009	0.691	< 0.001
		Right middle lobe	0.560	< 0.001	0.563	< 0.001
		Right lower lobe	0.575	< 0.001	0.700	< 0.001
Percentage of forced vital capacity	Absolute value of the logarithm of the Jacobian determinant	Whole lung	-0.361	0.023	-0.628	< 0.001
		Left upper lobe	-0.260	0.106	-0.587	< 0.001
		Left lower lobe	-0.397	0.011	-0.620	< 0.001
		Right upper lobe	-0.282	0.080	-0.565	< 0.001
		Right middle lobe	-0.150	0.354	-0.471	0.002
		Right lower lobe	-0.325	0.040	-0.630	< 0.001
	Absolute value of the warp-field magnitude	Whole lung	0.558	< 0.001	0.748	< 0.001
		Left upper lobe	0.450	0.004	0.563	< 0.001
		Left lower lobe	0.620	< 0.001	0.709	< 0.001
		Right upper lobe	0.558	< 0.001	0.668	< 0.001
		Right middle lobe	0.408	0.009	0.533	< 0.001
		Right lower lobe	0.572	< 0.001	0.667	< 0.001
Percentage of predicted forced expiratory volume in 1 s	Absolute value of the logarithm of the Jacobian determinant	Whole lung	-0.378	0.016	-0.615	< 0.001
		Left upper lobe	-0.365	0.021	-0.570	< 0.001
		Left lower lobe	-0.418	< 0.001	-0.605	< 0.001
		Right upper lobe	-0.379	0.016	-0.562	< 0.001
		Right middle lobe	-0.257	0.110	-0.459	0.003
		Right lower lobe	-0.350	0.027	-0.622	< 0.001
	Absolute value of the warp-field magnitude	Whole lung	0.556	< 0.001	0.771	< 0.001
		Left upper lobe	0.449	0.004	0.527	< 0.001
		Left lower lobe	0.609	< 0.001	0.727	< 0.001
		Right upper lobe	0.409	0.009	0.688	< 0.001
		Right middle lobe	0.556	< 0.001	0.527	< 0.001
		Right lower lobe	0.570	< 0.001	0.721	< 0.001
Percentage of diffusion capacity of the lung for carbon monoxide	Absolute value of the logarithm of the Jacobian determinant	Whole lung	-0.326	0.036	-0.618	< 0.001
		Left upper lobe	-0.302	0.058	-0.574	< 0.001
		Left lower lobe	-0.342	0.031	-0.608	< 0.001
		Right upper lobe	-0.250	0.119	-0.562	< 0.001
		Right middle lobe	-0.235	0.144	-0.461	0.003
		Right lower lobe	-0.384	0.015	-0.624	< 0.001
	Absolute value of the warp-field magnitude	Whole lung	0.557	< 0.001	0.714	< 0.001
		Left upper lobe	0.449	0.004	0.507	< 0.001
		Left lower lobe	0.611	< 0.001	0.679	< 0.001
		Right upper lobe	0.409	0.009	0.647	< 0.001
		Right middle lobe	0.556	< 0.001	0.461	0.003
		Right lower lobe	0.570	< 0.001	0.689	< 0.001
Serum KL-6 level	Absolute value of the logarithm of the Jacobian determinant	Whole lung	-0.228	0.157	-0.721	< 0.001
		Left upper lobe	-0.131	0.421	-0.572	< 0.001
		Left lower lobe	-0.258	0.108	-0.728	< 0.001
		Right upper lobe	-0.290	0.069	-0.681	< 0.001
		Right middle lobe	-0.131	0.422	-0.574	< 0.001
		Right lower lobe	-0.213	0.187	-0.737	< 0.001
	Absolute value of the warp-field magnitude	Whole lung	0.560	< 0.001	0.658	< 0.001
		Left upper lobe	0.443	0.004	0.470	0.002
		Left lower lobe	0.668	< 0.001	0.633	< 0.001
		Right upper lobe	0.381	0.015	0.624	< 0.001
		Right middle lobe	0.558	< 0.001	0.373	0.018
		Right lower lobe	0.574	< 0.001	0.642	< 0.001
CT-assessed disease severity	Absolute value of the logarithm of the Jacobian determinant	Whole lung	0.354	0.025	0.55	< 0.001
		Left upper lobe	0.318	0.045	0.612	< 0.001
		Left lower lobe	0.373	0.018	0.513	< 0.001
		Right upper lobe	0.368	0.020	0.657	< 0.001
		Right middle lobe	0.249	0.121	0.393	0.012
		Right lower lobe	0.347	0.028	0.481	0.002
	Absolute value of the warp-field magnitude	Whole lung	-0.543	< 0.001	-0.780	< 0.001
		Left upper lobe	-0.430	0.006	-0.694	< 0.001
		Left lower lobe	-0.615	< 0.001	-0.696	< 0.001
		Right upper lobe	-0.400	0.010	-0.620	< 0.001
		Right middle lobe	-0.530	< 0.001	-0.608	< 0.001
		Right lower lobe	-0.556	< 0.001	-0.697	< 0.001

*ADCT* Area-detector computed tomography, *KL-6* Sialylated carbohydrate antigen KL-6, *SD* Standard deviation

Table 5 demonstrates the results of the stepwise regression analysis of both indices among all lobes on supine and upright ADCT and pulmonary function test results, serum KL-6 level, and CT-assessed disease severity. Stepwise regression analyses on supine ADCT indicated that absolute values of the logarithm of the Jacobian determinant and the warp-field magnitude of the left lower lobe were the first and only step for pulmonary function test results, CT-assessed disease severity, or serum KL-6 levels of absolute value of the logarithm of the Jacobian determinant: 0.139 ≤ *r*^2^ ≤ 0.175, 0.007 ≤ *p* ≤ 0.018; absolute value of the warp-field magnitude: 0.371 ≤ *r*^2^ ≤ 0.447, *p* < 0.001. Conversely, according to the results of stepwise regression analysis on upright ADCT, the absolute values of the logarithm of the Jacobian determinant and the warp-field magnitude indicated that left lower lobe was the first step, and the right lower lobe was the second step for pulmonary function test results, except for %FVC, serum KL-6 level, and CT-assessed disease severity (absolute value of the logarithm of the Jacobian determinant: 0.503 ≤ *r*^2^ ≤ 0.674, *p* < 0.001 or 0.000 < *p* ≤ 0.006; absolute value of the warp-field magnitude: 0.474 ≤ *r*^2^ ≤ 0.652, 0.002 ≤ *p* ≤ 0.045). In addition, the absolute value of the warp-field magnitude on upright ADCT was determined as the first step, right lower lobe as the second step, and the right upper lobe as the third step for %FVC (*r*^2^ = 0.489, *p* = 0.041).

**Table 5:** Results of stepwise regression analyses of both indices among all lobes on supine and upright ADCT and pulmonary function test results, serum KL-6 levels, and CT-assessed disease severity

Pulmonary function test results, serum KL-6 level and CT-assessed disease severity	Index	CT system	Results of stepwise regression analysis				
			1st step	2nd step	3rd step	*r*^2^	*p*-value
Percentage of vital capacity	Absolute value of the logarithm of the Jacobian determinant	Supine ADCT	LLL	-	-	0.159	0.011
		Upright ADCT	LLL	RLL	-	0.545	0.002
	Absolute value of the warp-field magnitude	Supine ADCT	LLL	-	-	0.393	< 0.001
		Upright ADCT	LLL	RLL	-	0.652	0.002
Percentage of forced vital capacity	Absolute value of the logarithm of the Jacobian determinant	Supine ADCT	LLL	-	-	0.157	0.011
		Upright ADCT	LLL	RLL	RUL	0.489	0.014
	Absolute value of the warp-field magnitude	Supine ADCT	LLL	-	-	0.381	< 0.001
		Upright ADCT	LLL	RLL	-	0.595	0.006
Percentage of predicted forced expiratory volume in 1 s	Absolute value of the logarithm of the Jacobian determinant	Supine ADCT	LLL	-	-	0.175	0.007
		Upright ADCT	LLL	RLL	-	0.503	0.006
	Absolute value of the warp-field magnitude	Supine ADCT	LLL	-	-	0.371	< 0.001
		Upright ADCT	LLL	RLL	-	0.614	0.007
Percentage of diffusion capacity of the lung for carbon monoxide	Absolute value of the logarithm of the Jacobian determinant	Supine ADCT	LLL	-	-	0.147	0.014
		Upright ADCT	LLL	RLL	-	0.506	0.005
	Absolute value of the warp-field magnitude	Supine ADCT	LLL	-	-	0.373	< 0.001
		Upright ADCT	LLL	RLL	-	0.529	0.045
Serum KL-6 level	Absolute value of the logarithm of the Jacobian determinant	Supine ADCT	-	-	-	N/A	N/A
		Upright ADCT	LLL	RLL	-	0.674	< 0.001
	Absolute value of the warp-field magnitude	Supine ADCT	LLL	-	-	0.447	< 0.001
		Upright ADCT	LLL	RLL	-	0.474	0.045
CT-assessed disease severity	Absolute value of the logarithm of the Jacobian determinant	Supine ADCT	LLL	-	-	0.139	0.018
		Upright ADCT	LLL	RLL	-	0.614	0.007
	Absolute value of the warp-field magnitude	Supine ADCT	LLL	-	-	0.378	< 0.001
		Upright ADCT	LLL	RLL	-	0.546	0.034

*ADCT* Area-detector computed tomography, *KL-6* Sialylated carbohydrate antigen KL-6, *SD* Standard deviation

## Discussion

Our results demonstrate that the images obtained from upright ADCT may show even greater potential than supine ADCT in reflecting the respiratory functional alterations identified by pulmonary function tests. Hence, they may play a complementary role in quantitatively assessing the lung biomechanics of the whole lung and each lobe from paired inspiratory-expiratory CT data for pulmonary functional loss assessments and disease severity evaluations in patients with PPF. To our knowledge, no study has reported the potential utility of upright ADCT with quantitatively assessed lung biomechanics from paired inspiratory-expiratory CT data in PPF patients *versus* supine ADCT.

On comparing patients’ characteristics for each index determined from paired inspiratory-expiratory ADCT data between supine and upright ADCT in this study, there were no significant differences in pulmonary function test results, serum KL-6 levels, CT-assessed disease severity, absolute values of the logarithm of the Jacobian determinant, or the warp-field magnitude of whole lung or either lobe. These findings suggest that this study cohort had little variation from the above-mentioned parameters, although all PPF cases obtained with paired inspiratory-expiratory supine and upright ADCT were not the same patients.

Regarding the correlations between each index of the whole lung and each lobe from supine and upright ADCT with pulmonary function test results, serum KL-6 levels, and CT-assessed disease severity, upright ADCT showed significant and better correlations than supine ADCT on absolute values. Moreover, the absolute value of the warp-field magnitude showed better correlations with them than the absolute value of the logarithm of the Jacobian determinant in both CT systems. These facts were considered mainly due to the lung motions at scan position differences on supine and upright ADCTs, when assessing paired inspiratory-expiratory ADCT data in PPF patients.

The logarithm of the Jacobian determinant represented the local volume change at the voxel level. The voxel size after deformation is interpreted as unchanged if the value equals zero, as expansion if the value is positive, and as shrinkage if the value is negative. In contrast, the warp-field magnitude represents the magnitude of the movement of each voxel between the inspiratory and expiratory CT volumes. Therefore, regional movement was considered to be more sensitive than the local volume change for assessing the pulmonary function, predicting serum KL-6 levels, or evaluating CT-assessed disease severity in this setting.

When evaluating lung biomechanics in all lobes for influences on pulmonary functional loss and disease severity assessed by serum KL-6 level difference and CT-assessed disease severity, absolute values of the logarithm of the Jacobian determinant and the warp-field magnitude of the left lower lobe only affected them on paired inspiratory-expiratory supine ADCT. However, these indices in both lower lobes or the right upper lobe influenced them on paired inspiratory-expiratory upright ADCT. Furthermore, upright ADCT indices showed higher coefficients of determination than supine ADCT indices when applied to stepwise regression analyses between each index from all lobes and them. These findings suggested that upright ADCT gives us the opportunity for a more precise evaluation of lobe-based lung biomechanics than supine ADCT.

Several limitations associated with the present study warrant mention. First, clinically available upright ADCT systems have been installed in only a few institutions in Japan and are not widely available worldwide. Therefore, our study results only suggest the potential of upright ADCT compared to conventional supine ADCT in this setting. Furthermore, the CT system for upright ADCT is basically the same as that for the 3rd, 4th and 5th generations of ADCT, and hybrid-type IR and filtered back projection are applicable. In addition, some state-of-the-art radiation reduction and reconstruction algorithms, as well as sophisticated dual-energy CT techniques, are not available. Therefore, the application of the CT technique is limited compared to state-of-the-art ADCT systems as of 2024. Second, this study’s groups were too small to be trustworthy, even though both groups were balanced the characteristics of the patients. However, we could not directly compare changes in inspiratory-expiratory CT on upright and supine ADCT in the same patients because all CT data were retrospectively obtained from routine clinical examinations. Therefore, these facts may have been an obvious selection bias and confounding factor in this study. Moreover, although better correlations of upright ADCT rather than supine ADCT with pulmonary function test results were mainly due to different positions used during acquisition, these results did not mean that supine ADCT was not reflected by functional impairment. In addition, this study was a single-center study as well as vendor-specific technology, and the generalizability of the results is considered very limited. Third, we quantitatively assessed lung- and lobar-based biomechanics from paired inspiratory-expiratory ADCT data and correlated them with pulmonary function test results, serum KL-6 levels, and CT disease severity and did not compare diagnostic performance or influence on patients’ management or outcomes. Moreover, serum KL-6 level is a known marker for hypersensitive pneumonitis and the active phase of collagen vascular disease, but its relationship with disease severity in IPF was considered complex and did not always correlate directly [22–27]. In addition, most PPF patients in this study had nearly normal pulmonary function test results. Furthermore, no longitudinal or outcome data were compared between upright and supine ADCTs in this study. Fourth, this study was considered difficult to evaluate the reproducibility of this study by considering image registration validation in this study, although ICC of CT-disease severity between two investigators at each CT group was determined as excellent reliability [28]. Moreover, CT disease severity assessed in this study applied a visual scoring system, which had been used in several papers, to evaluate lung abnormalities. Moreover, a few investigator groups proposed other visual scoring systems [29–31]. Therefore, the score adopted in this study was a historically established tool for ILD evaluation, but it represents only one of several available systems, and more recently developed scores would be better applied in the future to further validate the results.

In addition, the external validations for quantitative and qualitative image analyses were not performed in this study. Therefore, the clinical relevance of upright ADCT has not been determined compared with conventional supine ADCT or other upright imaging tools [32–35], and further investigations with a large study population are warranted.

In conclusion, upright ADCT has equal to or better potential than supine ADCT and may play a complementary role for evaluating pulmonary functional loss and disease severity when paired inspiratory-expiratory ADCT is applied in patients with PPF.

## Data Availability

All data generated or analyzed during the current study are included in this published article.

## Abbreviations

ADCT: Area-detector computed tomography
ALAT: Associatión Latinoamericana de Tórax
ATS: American Thoracic Society
CT: Computed tomography
DL_CO_: Diffusion capacity of the lung for carbon monoxide
ERS: European Respiratory Society
FEV_1_: Predicted forced expiratory volume in 1 s
FVC: Forced vital capacity
ILD: Interstitial lung disease
IPF: Idiopathic pulmonary fibrosis
JRS: Japanese Respiratory Society
KL-6: Sialylated carbohydrate antigen KL-6
PPF: Progressive pulmonary fibrosis
VC: Vital capacity
